# Utilizing the Retrograde Descending Internal Mammary Vein in DIEP Flap Anastomosis

**Published:** 2018-10-29

**Authors:** Angie Zhang, Amra Kuc, Wilton Triggs, Deniz Dayicioglu

**Affiliations:** ^a^Morsani College of Medicine, University of South Florida, Tampa; ^b^Division of Plastic Surgery, Department of Surgery, Morsani College of Medicine, University of South Florida, Tampa

**Keywords:** venous congestion, chemotherapy catheter malposition, deep inferior epigastric perforator (DIEP) flap, breast reconstruction, additional venous drainage for DIEP flap

## DESCRIPTION

A 33-year old woman underwent a right mastectomy with bilateral deep inferior epigastric perforator (DIEP) flap reconstruction. Intraoperative discovery of a dislocated catheter prevented us from using the internal mammary vein (IMV) for anastomosis. With the aid of the venous congestion algorithm, we elected to anastomose the superficial inferior epigastric vein (SIEV) to the retrograde descending branch of the IMV stump.

## QUESTIONS

1. Which vessels are used in DIEP anastomosis?
2. What is the proposed algorithm to address intraoperative venous congestion?
3. What considerations were made in selecting vessels for anastomosis in our patient?
4. What is the anatomy of chemotherapy catheter placement and what are the rates of dislocation?

## DISCUSSION

Recipient vessels for microvascular anastomosis in DIEP flap breast reconstruction include the internal mammary, thoracodorsal, and circumflex scapular vessels. Of these 3, the IMV and the internal mammary artery (IMA) have diameters most compatible with those of deep inferior epigastric vessels and thus are the most commonly used recipient vessels.^1^ In selecting perforators, it is imperative to take into account the size of the accompanying vein to prevent venous congestion, which has an overall incidence of 2%. Venous congestion is reported to be the most common cause of flap loss.^2^

Venous drainage of the lower abdomen skin and subcutaneous tissue occurs primarily through the superficial venous system and secondarily through the deep venous system, with perforating veins interconnecting the 2 systems. When congestion occurs, assessed by the presence of an engorged and tense SIEV, a second anastomosis can be performed to ensure proper venous draining. An algorithm has been designed for choosing this second anastomosis.^3^ Anastomosis can be achieved by either connecting the SIEV to the deep inferior epigastric vein (DIEV) or by anastomosing the SIEV or DIEV to the distal retrograde IMV stump. The latter technique has been successful in relieving multiple cases of venous congestion. The relative lack of valves in the internal mammary system and alternative drainage through the proximal intercostal system may explain its success.

**Figure 1:**
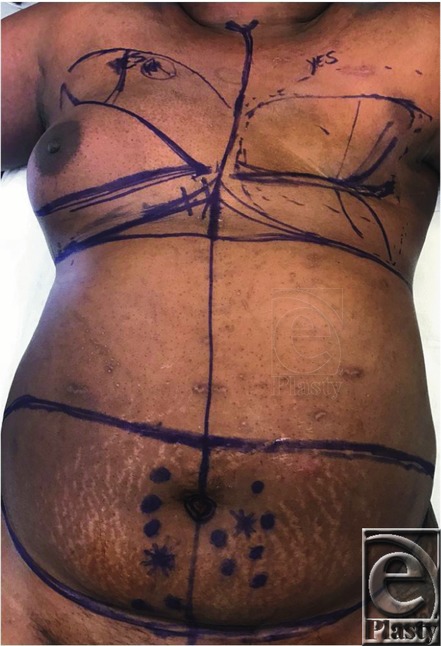
Preoperative view with markings.

**Figure 2:**
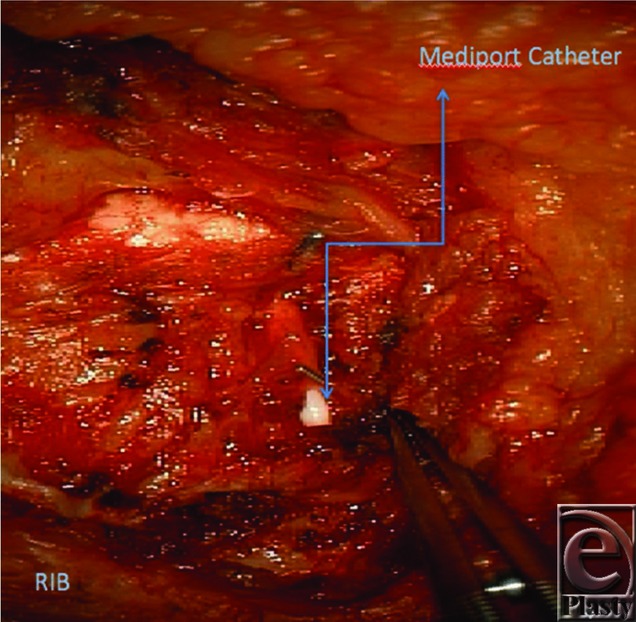
Mediport catheter within the right internal mammary vein.

**Figure 3:**
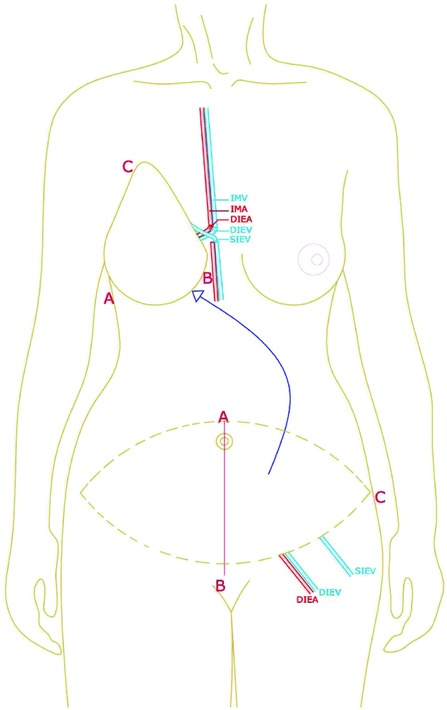
Illustration of anastomosis of the SIEV to the IMV stump due to thrombosis after port removal. The additional venous anastomosis was via the DIEV to the second IMV. SIEV indicates superficial inferior epigastric vein; IMV, internal mammary vein; DIEV, deep inferior epigastric vein; IMA, internal mammary artery; and DIEA, deep inferior epigastric artery.

**Figure 4:**
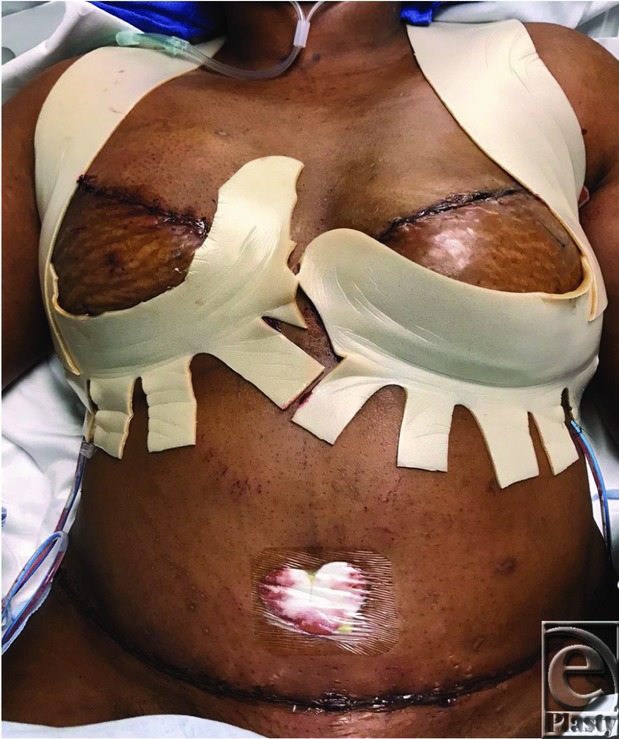
Postoperative view.

**Figure 5:**
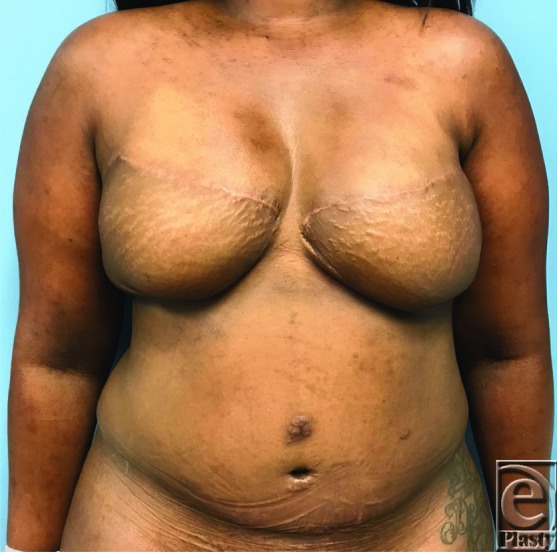
Three months postoperative.

We present the case of a 33-year-old female patient status post left mastectomy with chemotherapy and radiation therapy (Fig 1). With our team, she underwent a right mastectomy with bilateral DIEP flap reconstructions. The patient had bilateral chemotherapy port scars on her chest. First venous anastomosis included the DIEV to IMV with a 2.5-mm coupler. For arterial anastomosis, we chose the deep inferior epigastric artery to the IMA. We then discovered an incidental malposition of the Mediport chemotherapy catheter within the right IMV (Fig 2). Forgoing the availability of the right IMV for anastomosis due to thrombosis after port removal, we proceeded with connecting the SIEV to the retrograde descending branch of the IMV stump (Fig 3). Good flow through the flap was observed before and after closure (Fig 4). The patient recovered without complications (Fig 5).

Patients with breast cancer often require chemotherapy infusion, which can be administered by central venous catheters (CVCs). The preferred vein for a CVC is the right internal jugular vein (IJV) for its straight course to the right side of the heart and the lowest risk of venous stenosis and thrombosis.^4^ Spontaneous venous port migration is rare, occurring in about 1% to 4% of cases, but is a serious complication of long-term venous cannulation and is often asymptomatic.^5^ The most common migration site is the IJV, with other possible dislocations to the azygos vein, IMV, vertebral vein, subclavian vein, and brachiocephalic vein (BCV). In our case, the port was misplaced from the IJV to the IMV. Anatomically, the IMV travels adjacently, with its artery on the posterior aspect of the anterior chest wall to drain into the BCV, behind the sternal end of the clavicle and the first costal cartilage. Because the right IMV is often proximal to its origin, catheter dislocation to the right IMV can come from either BCV.^4^ Patients with highly moveable subcutaneous tissue secondary to pendulous breasts or obesity are the most susceptible to catheter dislodgement.^6^ Since the IMV is dilated by portal to systemic collateral circulation, patients with portal hypertension also have a higher risk of malposition.^7^

In the case of a known displaced catheter, we recommend preoperative imaging (chest computed tomography with contrast) to identify the catheter placement. With prior identification of the catheter or in an intraoperative discovery, such as in our case, following an intraoperative algorithm for the alleviation of venous congestion will help guide the surgeon in choosing the appropriate vessels for anastomosis.
